# Novel Principles and Methods in Bacterial Cell Cycle Physiology: Celebrating the Charles E. Helmstetter Prize in 2022

**DOI:** 10.3390/life13122260

**Published:** 2023-11-27

**Authors:** Vic Norris, Arieh Zaritsky

**Affiliations:** 1 Laboratory of Bacterial Communication and Anti-infection Strategies, EA 4312, University of Rouen, 76000 Rouen, France; 2 Life Sciences Department, Faculty of Natural Sciences, Ben-Gurion University of the Negev, Kiryat Bergman, HaShalom St. 1, Be’er-Sheva 8410501, Israel; ariehzar@gmail.com

This Special Issue celebrates the creation of the Charles E. Helmstetter Prize for Groundbreaking Research in Bacterial Cell Cycle Physiology. The Prize was inaugurated at the EMBO Workshop “Bacterial cell biophysics: DNA replication, growth, division, size and shape”, which was held in Ein Gedi, Israel, https://meetings.embo.org/event/22-bacteria-biophysics, accessed on 11–15 December 2022.

Understanding the cell cycle is fundamental to understanding the physiology of the bacterial cell and to all those fields that depend on it, ranging from clinical microbiology to industrial biotechnology, from microbial endocrinology to immunology, from the origins of life to xenobiology, and from environmental microbiology to synthetic biology. To take just one example, the cell cycle events of DNA replication and cell division underpin many approaches to dealing with the ever-growing threat of antibiotic resistance, including the phenotypic diversity that can lead to persister cells [1,2].

Our current understanding of the bacterial cell cycle owes a great deal to Charles Helmstetter, who has shaped the way microbiologists think about the bacterial cell for over fifty years. He has proved exceptionally gifted in formulating hypotheses for solving fundamental problems, in developing the experimental techniques to test them, in designing critical experiments, and in deriving paradigm-shifting ideas. In the early 1960s, well before the development of modern single-cell techniques, the main method of studying the bacterial cell cycle was through producing populations of cells belonging presumably to the same stage of the cell cycle—so-called synchronous cultures. The induction or selection methods used to produce such populations significantly perturbed the physiology of the cells and made it difficult to distinguish between real and artefactual changes in the cell cycle. Helmstetter’s primary motivation was to solve this problem. After several years of making and correcting errors, trying different approaches, and never giving up, he eventually invented the famous “Baby Machine”—a simple device continuously producing newborn bacterial cells at the earliest stage of the division cycle (most importantly, with minimal environmental changes) (memorialized in [3]). Years later, this method was adapted for eukaryotic cells (see below), and was complemented by the sophisticated “Mother Machine”, which combines microfluidics with fluorescence microscopy and single-cell analyses with big number statistics [4].

The Baby Machine possesses a feature that makes synchronous cultures unnecessary: the baby cells sequentially eluted from the machine are the descendants of cells in the parental culture in reverse age order, i.e., the first baby cells eluted are the products of the division of the oldest cells in the population. Using this ‘backwards’ method, the parental batch culture is pulse-labeled with radioactive thymidine before being transferred to the filter in the Machine; the radioactivity found in the eluted baby cells allows for the relative rate of DNA synthesis to be traced back to the division cycles of the cells growing in the unperturbed, steady state culture. The times of DNA replication’s initiation and termination can then be deduced from the stepwise rise or fall in the rate of incorporated thymidine, respectively. The results at different growth rates were puzzling; whilst the time between the termination of replication and subsequent cell division was relatively consistent, the time between initiation and termination was highly variable: initiation could occur at any stage of the cycle—even after termination! These findings were impossible to reconcile with the G1-S-G2 m sequence of the eukaryotic cell cycle paradigm.

This apparent paradox was resolved by Helmstetter and Steve Cooper in 1968 by their paradigm-shifting proposal of the *I* + *C* + *D* sequence for the bacterial cell cycle. Here, *C* is the chromosome replication time, *D* is the time between termination of replication and completion of division, and *I* is the time between consecutive initiations (equal to, but not coinciding with, the inter-division time, *τ*). Three major processes are responsible for a newborn cell growing and dividing to yield two daughters: the continuous increase in biomass (i.e., growth) and the discrete, cell cycle processes of chromosome replication (which is bounded by initiation and termination) and cell division. Helmstetter and Cooper considered these cell cycle processes to be highly coordinated and to overlap, albeit partly independently. Their proposed model solved the paradox of how a dynamic steady-state growing population can maintain a constant size distribution and yet have a mass doubling time *τ* that is shorter than the time it takes a cell to replicate its chromosome and then divide (*I* < *C* + *D*). Furthermore, if cells divide at the same rate as their mass increases, but *C* is longer (*I* = *τ* < *C*), daughters that lack DNA may be created. These results led them to conclude that rounds of replication can also overlap so that the inter-initiation time *I* is shorter than *C*, consistent with the observed multi-forked replication. Once termination occurs, there are effectively two separate termini, i.e., chromosomes whose replication continues. Hence, these two structures in which replication is ongoing can be separated and segregated into the daughter cells, a fundamentally different picture from what happens in eukaryotes. The main characteristics of the bacterial cell cycle can be understood and predicted quite correctly for a wide range of values of *C*, *D* and *I* (=*τ*), as well as for the various transitions between them, as is clarified by the *I* + *C* + *D*-based simulation program [5].

A few examples of subsequent work on the cell cycle by Helmstetter and his collaborators are briefly summarized here. In 1986, Leonard and Helmstetter reported that *oriC* plasmids (mini-chromosomes) initiated replication in coordination with the chromosome, irrespective of their copy number. With Olga Pierucci, he obtained the first evidence that chromosome segregation is not random. He and Leonard proposed an explanation for the observed non-random segregation pattern based on a mechanism that distinguishes between template strands of different ages. In 1997, he and Bogan found that the transcription of genes in the vicinity of *oriC*, namely *mioC*, *gidA* and *dnaA*, was affected by sequestration at the level of initiation rather than of elongation. Helmstetter and collaborators also found that transcription of several genes involved in the cell cycle, *ftsZ*, *dam*, *nrdA*, *mukB* and *seqA*, was reduced at a certain stage during chromosome replication, apparently coincident with the time the genes replicated. As part of a series of studies on plasmid replication, he and Leonard showed that F plasmid replication is not confined to a period of the cell cycle. In 1992, he and Zaritsky found that division was delayed after a nutritional shift down, and then its rate maintained for an extended period; these studies were followed up by other collaborators. With Thornton and Edward, he adapted the Baby Machine technique for cell cycle studies on hematopoietic mouse and human cells, and with collaborators, analyzed transcriptomics of cyclins and PCNA. Finally, in 2008, he used BrdU incorporation to study time zones of replication in the entire mouse genome.

It is well known that just as the importance of a prize enhances the reputation of its recipients, so the reputation of its recipients enhances the reputation of the prize. This is indeed the case for the Charles E. Helmstetter Prize, the first awardees of which are three distinguished specialists: Philip C. Hanawalt, Elio Schaechter, and Conrad L. Woldringh. Had the prize been named after someone else, Helmstetter himself would have been a prime recipient! We strongly advise those interested in the cell cycle to listen to their videos and read their papers in this Special Issue. Moreover, many of the other papers in this issue describe the contributions made by those who can fairly be said to have founded the bacterial physiology and the cell cycle field as we now know it (Figure 1).

The study of DNA repair and the cell cycle in bacteria has direct implications for human health. Hanawalt details his discovery that the bacterial DNA replication cycle can be synchronized by temporarily inhibiting RNA and protein synthesis, and that an RNA synthesis step is required for the initiation but not for the completion of chromosome replication. His earlier graduate studies on the effects of UV on macromolecular synthesis in bacteria led to his discovery of repair replication and the co-discovery of excision-repair, which requires an undamaged complementary DNA strand. He postulates that excision-repair was essential for the persistence of life from its earliest forms. In summary, this paper offers insight into how fundamental discoveries can be made with relatively simple bacterial systems.

Schaechter [6] reminisces about his time in the laboratory of Ole Maaløe and his experience of the ‘Copenhagen School’, which was characterized by systematic approaches and careful measurements. This led to the discovery of the direct relationship between cell size and growth rate afforded by medium composition, and the constant rates of macromolecular syntheses in all media (at a given temperature). Back in America, he continued to make major discoveries about polysomes, and about the attachment of the chromosome to the membrane. Schaechter’s discoveries have structured microbiology.

Woldringh [7] invokes polymer physics to help answer the question as to how bacteria segregate their chromosomes; in his hypothesis, the newly synthesized daughter strands, which are proposed to exist already as separate blobs in the early replication bubble, expand into large domains of the left and right chromosome arms, flanking the origin. One attractive advantage of this idea is that segregation only requires de novo DNA synthesis (though other players, like SMC-like proteins, are not excluded).

Donachie [8] asks “Why are *Escherichia coli* cells the size and shape that they are?” This is basically the question that he himself was asked by the late Kurt Nordström, many years ago. His paper argues that cell size and the geometry of cell growth are adapted to fit a growth rate-independent constant: the separation distance (“Unit Length”) between sister nucleoids after the completion of each round of chromosome replication. He also suggests that (in *E. coli* and related Gram-negative rods) the mechanism of separation of sister chromosomes may be set by physics, not primarily by genes.

The metabolic control of replication couples the initiation and elongation phases of DNA replication to growth rate affected by nutrient richness; in their paper, Holland et al. provide evidence that this control, which is fundamental to genetic stability, depends on the dynamic recruitment of the glycolytic enzyme PykA by DnaE at sites of DNA synthesis.

Nanninga [9] gives his recollections of the origins of electron microscopy at Amsterdam University, and the importance of freeze-fracturing. He relates how the study of the cell cycle used electron microscopy to analyze bacteria grown in a steady state, and to show that the zonal growth of the envelope could not be responsible for the segregation of envelope-attached DNA. The combination of bacterial physiology, electron microscopy, and image analysis has been termed the Amsterdam School by Arthur Koch on several occasions. This work, among others, led to the idea that PBP3-independent peptidoglycan synthesis preceded a PBP3-dependent step. His experience with the ‘mesosome’ is instructive, and his review should be obligatory reading for those interested in cell division. Nanninga briefly describes the pioneering efforts to construct a confocal scanning light microscope in Amsterdam.

Kohiyama [10] tries, in his words, “to throw a cobblestone in the pond” (from the French ‘jeter un pavé dans la mare’). He and his colleagues revisit the DnaA story from the point of view of hyperstructures; they propose the existence of a physico-chemical clock that simultaneously triggers the initiation of chromosome replication and of cell division.

Leonard [11] recounts his experience as a postdoctoral fellow with Helmstetter, focusing on construction of mini-chromosomes and using the ‘backwards’ baby machine to study their replication timing. He describes how his trials and tribulations eventually led to important discoveries about how mini-chromosomes replicate in synchrony with the host origin, how they segregate into daughter cells, and the important role of DNA supercoiling in mini-chromosome function. He discusses his experiences studying plasmid replication during the cell cycle.

Liu and his group [12] show the disconcerting extent to which the type of software and the values of settings can affect the quantification of cell size parameters using microscopic images. They argue persuasively that microscope-independent methods should be used to validate conclusions and provide a precious illustration in the case of the initiation mass. This caveat-focused paper should be read by all those interested in the cell cycle.

Finally, Zaritsky’s review [13] summarizes his own contributions to the field, mostly related to the demonstration that DNA replication rate can be manipulated in thymine auxotrophic mutants by the external thymine concentration supplied, thus dissociating it from the rate of cell mass growth and duplication. This quantitative description complements Helmstetter’s findings that in thymine prototrophs the replication time *C* is constant under a wide range of doubling times *τ*, as determined by the medium composition. This seemingly simple physiological manipulation enabled Zaritsky to discover the existence of Eclipse, namely a minimal distance possible between successive replisomes, which during thymine limitation causes the otherwise constant mass at initiation to gradually increase. Furthermore, cell width was found to be tightly related to nucleoid complexity (*NC*), which is the amount of DNA in genome equivalents associated per *terC* (i.e., the chromosome terminus). *NC* depends only on the number of replication positions *n*, where *n* = C/*τ*, and can therefore be varied by changing *τ* or *C*.

## Figures and Tables

**Figure 1:**
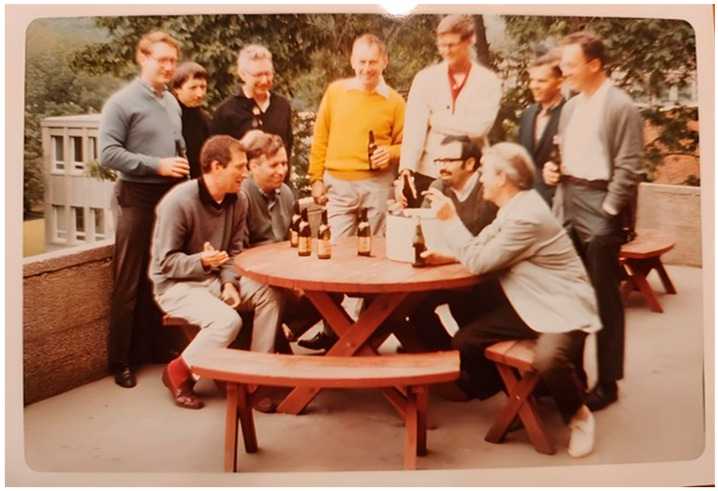
Several scientists, among the founders of bacterial physiology and the cell cycle, at the Cold Spring Harbor Symposium on Quantitative Biology, “Ole Maaløe with Alumni”, 1968. Front Row (sitting, Left-To-Right): KG Lark, M Schaechter, S Cooper, O Maalϕe, Back Row: PC Hanawalt, DJ Clark, C Levinthal, J Watson *, P Kuempel, CE Helmstetter, D Glaser **. Nobel Prize Laureates in: * Physiology/Medicine (1962); ** Physics (1960) (From the personal collection of Philip C Hanawalt; https://web.stanford.edu/~hanawalt/), accessed on 22 November 2023. **“If I have seen further, it is by standing on the shoulders of giants”**—Sir Isaac Newton.
